# Prediction of Ablation Index and Lesion Size Index for Local Impedance Drop-Guided Ablation

**DOI:** 10.3390/jcm14030832

**Published:** 2025-01-27

**Authors:** Lukas Sprenger, Fabian Moser, Vera Maslova, Adrian Zaman, Marc Nonnenmacher, Sven Willert, Derk Frank, Evgeny Lian

**Affiliations:** 1 Department of Internal Medicine III (Cardiology and Intensive Care Medicine), University Hospital Schleswig-Holstein (UKSH), Arnold-Heller-Str. 3, 24105 Kiel, Germany; 2 German Centre of Cardiovascular Research (DZHK), Partner Site Hamburg/Kiel/Lübeck, Arnold-Heller-Str. 3, 24105 Kiel, Germany

**Keywords:** atrialfibrillation (AF), pulmonary vein isolation (PVI), radiofrequency ablation (RFA), ablation index (AI), lesion size index (LSI), local impedance (LI)

## Abstract

(1) **Background**: The effectiveness of RF ablation for PVI depends on the lesion location and size to achieve continuous and durable lesion lines. AI and LSI are widely accepted lesion metrics for guiding the ablation procedure. LI dynamics is another parameter that guides PVI and does not rely on input variables. Limited data are available on a direct comparison between lesion metrics. Our study aims to compare RF application durations and influencing factors during index-guided (AI and LSI) and LI-guided approaches by predicting lesion metrics using machine learning. (2) **Methods**: While the coefficients in AI and LSI formulas are not disclosed, we trained custom machine-learning models based on Random Forest and Gradient Boosting Regressors to predict AI and LSI metrics for LI-guided ablations. (3) **Results**: The median RF application durations differed significantly between the lesion metrics, with 7.32, 19.91, and 11.92 s for AI-, LSI-, and LI-guided procedures, respectively. Mean CF was found to be an important predictor of RF application duration for the AI- and LSI-guided approaches. (4) **Conclusions**: Depending on the metric used, the significant differences in RF application durations suggest that an AI-guide approach may allow for shorter RF application durations, followed by LSI-guided and LI-guided procedures. Further studies are needed to evaluate the safety and efficacy of these results in a clinical setting.

## 1. Introduction

Radiofrequency (RF) catheter ablation is an established treatment option for atrial fibrillation (AF) [1]. Ablation success highly depends on the location and size of the lesion created by the ablation catheter, thereby leading to a continuous and durable lesion set forming an ablation line between the pulmonary vein (PV) and the left atrium. The main reason for AF recurrence after ablation is the electrical reconnection of the PV and the left atrium [2], whereas prolonged RF application duration bears the risk of complications, such as steam pops, pericardial effusion, or atrioesophageal fistula [3]. Aiming at creating durable ablation results while mitigating complications emphasizes the importance of precise lesion size prediction.

The Ablation Index (AI) and Lesion Size Index (LSI) are widely accepted, easy-to-use lesion metrics for estimating the RF lesion size and help guiding the ablation procedure. Depending on the physicist’s discretion, they are available in their corresponding 3D mapping systems: CARTO© 3 (Biosense Webster, Irvine, CA, USA) and EnSite™ (Abbott, Abbott Park, MA, USA). The feasibility of both AI and LSI has been shown in previous studies [4,5,6]. Both metrics are dependent on their input parameters, such as contact force (CF), delivered power (*P*), radiofrequency current (*I*), and application duration (τ). Local impedance (LI) is another parameter that guides catheter ablation and does not depend on further input parameters. LI dynamics measurement was implemented in the RHYTHMIA HDx™ (Boston Scientific, Marlborough, MA, USA) platform with DIRECTSENSE™ technology. The LOCALIZE trial showed that LI dynamic predicts acute and chronic PV segment conduction block, even without CF monitoring [7]. Next-generation ablation catheters (IntellaNav Stablepoint™ with DIRECTSENSE™ technology) integrate both LI and CF measurement, but the current RHYTHMIA HDx™ platform neither provides AI nor LSI indices to guide the ablation procedure.

Currently, there are a lack of data on the direct comparison of lesion metrics, such as AI-, LSI-, and LI-drop-guided ablations. Most studies have reviewed lesion metrics individually regarding their acute efficacy and long-term success. For example, they show shorter procedure times and fewer PV gaps for an LSI-guided approach than an LI-drop-guided ablation procedure [8]. However, a direct comparison regarding RF application duration and influencing factors for lesion creation is particularly required.

Our study aims to provide insights into RF application durations and influencing factors by comparing index-guided (AI and LSI) and LI-guided approaches, which is achieved by predicting AI and LSI metrics using a machine learning approach and by showing the feasibility of their applicability in a clinical setting.

## 2. Methods

While CF and LI monitoring (Stablepoint™, RHYTHMIA HDx™ with DIRECTSENSE™ technology, Boston Scientific, Marlborough, MA, USA) are available simultaneously during ablation, AI and LSI formulas contain proprietary coefficients, which are not disclosed (see Equations (1) and (2)).

(1) $$\mathrm{Ablation}\;\mathrm{Index}={\left\{K\cdot \int_{0}^{\tau }CF_{\left(\tau \right)}^{a}\;P_{\tau }^{b}\;d\tau \right\}}^{c},$$

(2) $$\mathrm{Lesion}\;\mathrm{Size}\;\mathrm{Index}=b_{0}\left(1-e^{\frac{-F}{b_{1}}}+b_{2}\right)\left(1-e^{\frac{-I^{2}}{b_{3}^{2}}}\right)\left(1-b_{4}+\frac{b_{4}\left(1-e^{\frac{-\tau }{b_{5}}}\right)}{1-e^{\frac{-60}{b_{5}}}}\right).$$

Equation (1) shows how the Ablation Index (AI) is calculated, while Equation (2) shows how the Lesion Size Index (LSI) is calculated. Ablation Index (AI, arbitrary units): CF = contact force; *P* = power; τ = application duration; and K,a,b,c represent constants (proprietary data). Lesion Size Index (LSI, arbitrary units): *F* = 6 s sliding window of average contact force; *I* = 6 s sliding window of average radiofrequency current; τ = time; and $b_{0},b_{1},b_{2},b_{3},b_{4},b_{5}$ represent constants (proprietary data).

We performed a two-step approach to estimate the AI and LSI for the lesions created by the RHYTHMIA HDx™ system, as these indices are not provided by the system. In the first step, as predicting lesion indices can be comprehended as a regression problem, we trained two machine learning algorithms for AI and LSI predictions using collected ablation data. In the second step, we retrospectively analyzed the atrial ablation data of patients undergoing LI-drop-guided ablation, utilizing our pre-trained models to predict AI and LSI lesion metrics.

Data preparation, model generation, and statistics were conducted in Python using Numpy [9], Pandas [10], scikit-learn [11], Scipy [12], Statsmodel [13], pyGAM [14], and Miceforest. Matplotlib [15] and Seaborn [16] were used for visualization. For the software library version used, see Table A1.

### 2.1. Patient Population

Patient data, both for model generation and data analysis, were provided and anonymized at the time of export from its specific ablation system, with baseline patient characteristics attached. For model generation, patients undergoing radiofrequency PVI for the ablation of paroxysmal or persistent AF using either AI-guided (Carto© 3 system, Biosense Webster, Irvine, CA, USA) or LSI-guided (EnSiteX™ system, Abbott, Abbott Park, MA, USA) approaches were selected. Data were exported from the RHYTHMIA HDx™ system with DIRECTSENSE™ technology for data analysis.

All procedures were carried out in accordance with the current guidelines [1]. SmartTouch^®^ and TactiCath™ ablation catheters were used for index-guided ablation while using the CARTO© 3 system (Biosense Webster, Irvine, CA, USA) and EnSite™ system (Abbott, Abbott Park, MA, USA) accordingly. The energy was delivered using an IntellaNav Stablepoint™ ablation catheter (RHYTMIA HDx™ system, Boston Scientific, Marlborough, MA, USA) for the LI drop-guided procedures.

### 2.2. Model Generation

We gathered two training datasets by exporting the ablation data from ten patients that had been undergoing ablation for paroxysmal or persistent atrial fibrillation from their respective mapping systems. This resulted in a total of 553 (AI) and 22 (LSI) lesions. As we aimed to be able to predict the lesion indices for every time point τ during the ablation process, we further separated the independent and dependent variables during lesion creation, with a total of 529,684 (AI) and 42,538 (LSI) data points for training purposes. Furthermore, using the Simpsons’ rule [17] during data preparation, we calculated the force–time integral for every time point τ during lesion creation. For the parameter space of both models, refer to Table 1 and Table 2.

We used the Shapiro–Wilkins and Anderson–Darling tests as normality tests. For both training datasets, the Shapiro–Wilkins and Anderson–Darling tests indicated a non-Gaussian distribution. Additionally, the variance inflation factors (VIFs) and correlation coefficients suggested the existence of multicollinearity, potentially affecting the stability of the models. To address these challenges, we compared a Random Forest Regressor [18,19] and a Gradient Boosting Regressor [19] alongside three linear models—Ridge, Lasso, and ElasticNet [19]. During the training of the LSI model, we employed quantile transform to handle the non-Gaussian distribution and skewness of the LSI training data.

Furthermore, we conducted hyperparameter [20] tuning to improve the performance of all models using *GridSearchCV* with a 10-fold cross-validation. We chose the hyperparameters to be optimized based on our own conducted early experiments to account for both simple and more complex models. For the optimized hyperparameters and their value ranges, refer to Table A2.

To evaluate our final models, we used the coefficient of determination ($R^{2}$) and the mean absolute error (MAE) while applying a 30% split for cross-validation. Regarding our AI prediction model, the Random Forest model performed best with an $R^{2}$ of 0.85 and an MAE of 6.62. For our LSI prediction, we chose the Gradient Boosting model, as it showed the best performance with an $R^{2}$ of 0.97 and an MAE of 0.10. The three linear models also performed less successfully, most likely due to their inability to model the non-linear relationship of the underlying data (refer to Table 3 for the complete test results).

### 2.3. Data Analysis

**Figure 1:**
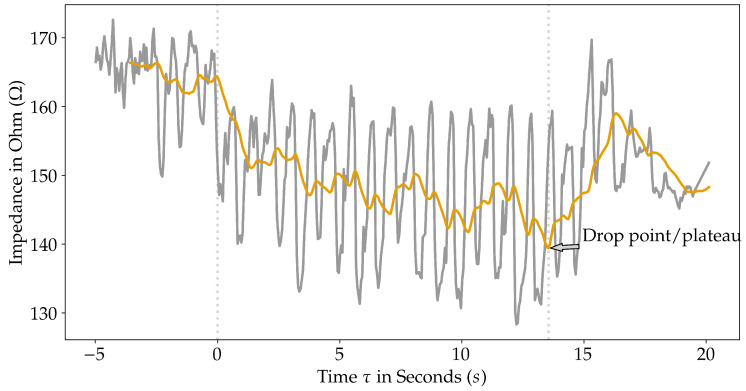
According to the LOCALIZE trial, raw LI measurements were first filtered through a moving mean filter with a window length of 1.5 s [7]. The figure shows both raw (grey) and filtered LI (orange), as well as the LI drop point/plateau. Data integrity was ensured by trimming the left edge of the filtered LI data. This was due to using a moving mean filter extending over the initial data points with a window size of 1.5 s.

Subsequently, we analyzed the atrial ablation data of 27 patients who underwent LI-drop-guided ablation. For patient demographics, refer to Table 4. To our knowledge, no patients suffered from procedural complications or had to undergo a redo procedure. The data exported from the RHYTHMIA HDx™ mapping system (Boston Scientific, Marlborough, MA, USA) contained raw LI, CF, and power measurements. We used linear interpolation to transfer to a standard time base and sampling frequency of 997 Hz for every ablation trace. According to the LOCALIZE trial’s approach, raw LI measurements were first filtered through a moving mean filter with a window length of 1.5 s (see Figure 1) [7]. We based all further calculations on the filtered LI, which we refer to as LI in the following sections. In analogy to Ohm’s law for alternating current, the RF current was calculated from the LI and power measurements.

The data contained three-dimensional coordinates of the ablation catheter’s tip location. Using the k-nearest neighbor algorithm [21,22], we calculated the linear interlesion distance (ILD) between two adjacent lesions. Therefore, the tip’s mean position was calculated for each lesion.

**Figure 2:**
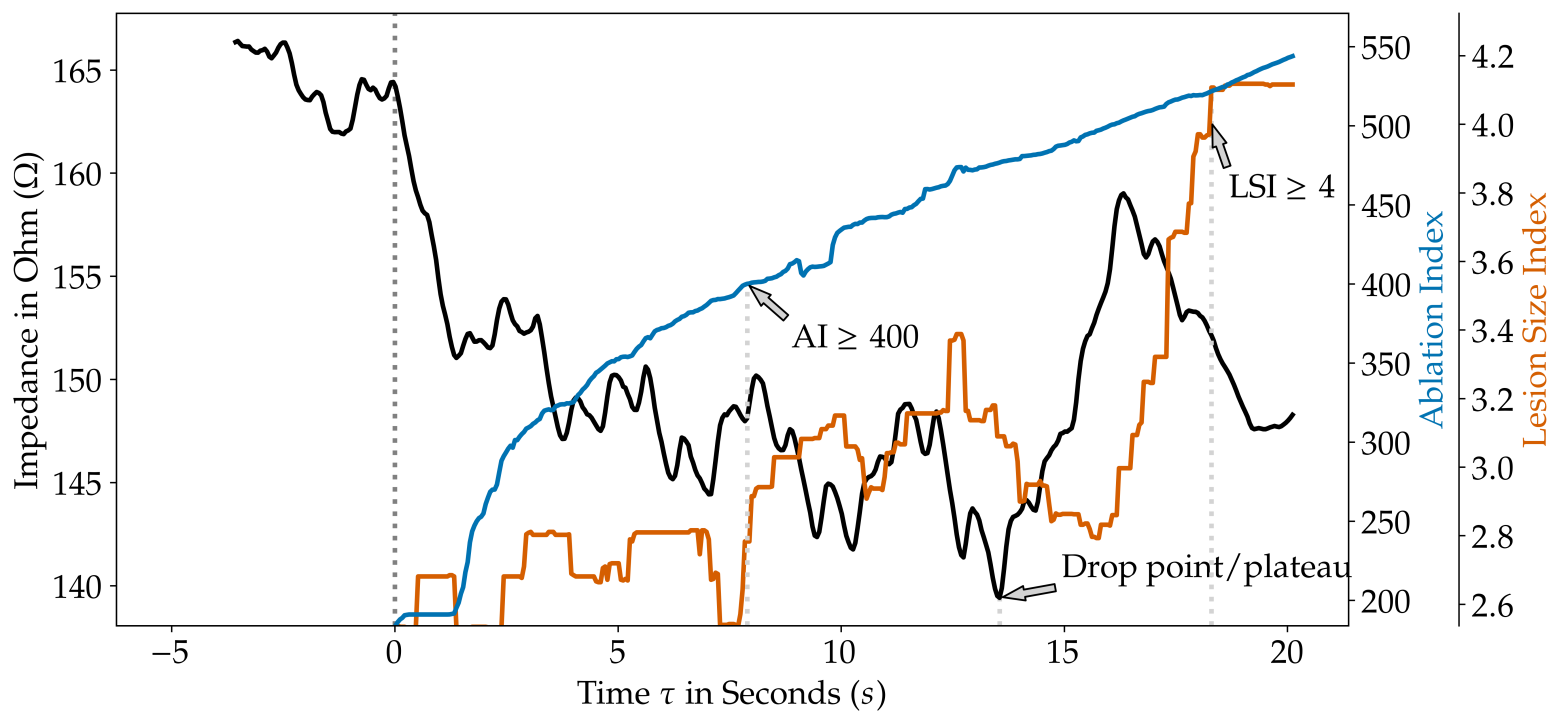
The filtered local impedance (LI), the predicted Ablation Index (AI), and the predicted Lesion Size Index (LSI) during an ablation procedure for every time point τ. The arrows mark the AI- and LSI-targets and the LI drop plateau. Note the AI and LSI prediction start for time points was τ≥0 seconds (s). The LI drop plateau was defined as the local minimum. While only lesions that reached the operator’s desired LI-drop target were exported, we visually confirmed a correctly set LI drop point/plateau for each lesion.

We then calculated the predicted indices for every time point τ on the ablation trace using our earlier trained AI and LSI prediction models (Figure 2). The local impedance drop plateau was defined as the local minimum. While only lesions that reached the operator’s desired LI-drop target were exported, we visually confirmed a correctly set LI drop point/plateau for each lesion. AI targets were set to ≥400, and LSI targets were set to ≥4.

Lesion metrics were then exported for further statistical analysis.

### 2.4. Statistics

During data preparation, we excluded lesions with missing values for the AI prediction model. We used Little’s MCAR (Missing Completely at Random) test to address missing values for our LSI prediction model. With the test yielding a chi-square statistic of 12.93 and a *p*-value of 0.166, we employed Multiple Imputation by Chained Equations (MICE) with five iterations to handle the missing values for the LSI ablation duration. Subsequently, to further provide robustness to our results, a sensitivity analysis of the imputation process was performed. We compared MICE against the mean, median, and k-nearest neighbor (KNN) imputations with consistent results of 0.802 for $R^{2}$ and 0.974 for MAE. Also, we tested for different iteration counts for MICE (5, 10, and 15), with stable performance metrics ranging from 0.713 to 0.748 for $R^{2}$ and 1.078 to 1.130 for MAE. We, therefore, opted to use MICE as our imputation method for the LSI duration data. Additionally, lesions with RF application durations over 30 s and ILD over 6 mm were excluded beforehand according to the CLOSE-Protocol [4] and Kanamori et al. [23]. Overall, a total of 13% of all lesions were excluded.

We reported continuous variables as the median with inter-quartile range (Q1–Q3) or mean ± standard deviation, and the categorical variables were summarized as count and percentage. We tested against a Gaussian distribution using Shapiro–Wilkins and Anderson–Darling tests. In addition, we utilized Q-Q plots and histograms for visual evaluation.

The Friedman and post-hoc Wilcoxon signed-rank tests were used to compare the ablation durations between groups.

The residuals were not normally distributed, and the Lagrange Multiplier test indicated signs of heteroscedasticity. Since the Gauss–Markov assumptions were violated, we used Generalized Additive Models (GAMs) to capture the potential non-linear relationships and Random Forests as a robust, non-parametric approach to regression analysis.

## 3. Results

Our main findings are presented in Table 5. The results indicate that the median RF application duration differed significantly depending on the lesion metric used (Friedman-Test: $\chi^{2}\left(2\right)=1418.95$, p≤0.001, and n=1090). While the median RF application duration guided by an AI target of ≥400 was 7.32 (IQR = 5.05–9.57) s, the median RF application duration guided by an LSI target of ≥4 was 19.91 (IQR = 17.59–22.95) s. The LI drop plateau was reached after a median RF application duration of 11.92 (IQR = 8.02–16.72) s.

There is a moderate difference when comparing an LI-guided approach and an AI-guided approach in significantly shorter RF application durations for AI-guided procedures, W=116,012.00, $p_{Bonferroni}<0.001$, *r* (Cohen, 1992) = −0.53. In contrast, the LI plateau is achieved more quickly than an LSI target of ≥4, W=31,720.00, $p_{Bonferroni}<0.001$, *r* (Cohen, 1992) = −0.77. Comparison of the AI-guided and LSI-guided RF application durations also revealed a substantial difference with shorter RF application durations for AI guided procedures, W=116.00, $p_{Bonferroni<0.001}$, *r* (Cohen, 1992) = −0.87.

In predicting the RF application duration for an AI-guided procedure, GAMs and Random Forests consistently identified the mean CF, starting CF, LI drop, and LI starting impedance as critical predictors. While all parameters were significant (GAMs ps<0.001), the mean CF had the highest importance (0.811) in prediction RF application duration, followed by starting CF (importance 0.110). In contrast, in an AI-guided approach, ILD had no predictive value for RF application duration.

Regarding the predictors for the RF application duration in LSI-guided procedures, the mean CF, LI start, and drop values were significant factors influencing the RF application duration (GAMs ps<0.001). However, their importance differed, with the mean CF values having the highest importance (importance 0.617), as identified by Random Forest. Contrary to an AI-guided approach, the interlesion distance was a significant (GAMs ps<0.001) factor; however, it was a less important factor (importance 0.067) than the mean CF in predicting RF application durations.

In contrast to AI- and LSI-guided procedures, GAMs and Random Forest identified the LI drop and LI start values as significant (GAMs ps<0.001) and important (0.325 and 0.195) predictors, respectively, for RF application duration in LI-guided procedures. While GAMs and Random Forest identified significant and important predictors, they were put into perspective by emphasizing the Random Forest metrics. As the mean squared error was considered high (mean CV MSE = 38.432) and the coefficient of determination ($R^{2}=0.064$) was low, the Random Forest model was less reliable, possibly compromising the identified values of the predictors.

## 4. Discussion

This study provides new insights into the level of lesion creation during catheter ablation, showing significant differences in the RF application durations between the AI-, LSI-, and LI-guided ablation strategies with AI and LSI targets set to 400 and 4, respectively. The data analysis based on our trained machine learning models predicted the AI and LSI values and, thus, the presented data are based on our models’ reliance in the context of machine learning. The AI and LSI metrics remain proprietary to their respective mapping systems. This retrospective cohort study with a comparative analysis is the first to provide a direct comparison to such an extent.

With RF catheter ablation already providing an established treatment option, especially in AF, to an aging population in Europe and the consecutively rising burden of arrhythmias for individuals, as well as the healthcare system, its importance will increase. While recent analysis, such as the study conducted by Wita et al., shows regional differences in ablation rates [24], it is essential to understand and compare the current metrics to optimize outcomes across different healthcare systems.

Published data showed longer RF application durations in LSI-guided procedures compared to LI-guided procedures, which might result in more durable lesions [8]. Regarding RF application duration and energy delivery, HPSD ablation is currently being reviewed [3,25], with recent studies, such as La Fazia et al., showing the impact of the power setting in achieving transmural lesions and their effect on arrhythmia recurrence [26].

This current study had its main focus on the level of lesion creation. We report significant differences in the RF application duration between AI-, LSI-, and LI-guided approaches. The significantly shorter RF application duration observed with an AI-guided ablation approach compared to an LSI-guided approach was one of the most notable outcomes. Similarly, the RF application duration for an LI-guided approach was significantly shorter than an LSI-guided approach. While the exact clinical impact of different RF application durations remains unclear, our findings may suggest that shorter RF application durations with an AI-guided approach lead to shorter, more efficient procedures while mitigating complications such as atrio-esophageal fistula. On the other hand, LSI-guided ablations with longer RF application durations may subsequently result in more durable lesions, as suggested by Lian et al. [8].

Predictors for RF application durations vary between approaches, with the mean CF having the highest relative importance for AI- (0.811) and LSI-guided (0.617) approaches. As the AI formula already incorporates CF as one input variable and the LSI formula contains a 6 s sliding window of mean CF, the finding of the mean CF as the most important predictor does not seem to have much novelty. However, this proves that our machine learning model predictions work as expected, as AI and LSI rely on their input variables. Furthermore, the results underline the necessity of stable tissue–catheter contact for durable lesions. However, the variability in CF between cases may depend on anatomical differences, such as atrial wall thickness, or operator techniques.

LI drop and starting LI values were identified with regression analysis as important predictors (0.325 and 0.195) for RF application duration during LI-guided procedures. These results are in line with the findings of the LOCALIZE trial [7] while also linking them with RF application duration. The results of the LOCALIZE trial [7] may provide a reference for our machine learning models as the findings of influencing factors are assumed to be of close similarity. In contrast to the LOCALIZE trial [7], the relative importance of LI drop and starting LI as a predictor for RF application duration when compared to the mean CF for AI- and LSI-guided procedures, as a predictor, showed a considerably lower predictive performance. This suggests that LI, as a real-time indicator, lacks the ability to fully capture the complexity of lesion creation. The Random Forest model’s lower predictive performance ($R^{2}=0.064$) further underlines these findings as it may influence the operator’s choice of metric to guide the procedure.

More studies are needed to evaluate the safety and efficacy of these results in a clinical setting. Assessment of different machine learning techniques and the incorporation of more procedural and periprocedural parameters will help to construct a more patient-tailored ablation approach [27,28,29].

### Study Limitations

Our study has several fundamental limitations that primarily arise from using a two-step approach. First, the limitations that apply when using machine learning. Second, the limitations arise from methodological and underlying data challenges.

While the AI and LSI metrics are proprietary, the exact formula coefficients have not been publicly disclosed. Thus, our approach relies on our models correctly predicting their according indices. Although we used Random Forest and Gradient Boosting for our underlying models, the small training data size of 52,968 (AI) and 4253 (LSI) for every time point τ during lesion creation can lead to overfitting. In particular, this is problematic when applying lesion metric prediction to unseen data. However, Random Forest is less susceptible to overfitting due to its nature of reducing variance compared to Gradient Boosting. This especially applies to a limited training data parameter space. On the other hand, Gradient Boosting reduces bias, thus leading to a better fit within the parameter space. Regarding outliers, Random Forest tends to be more resistant as the averaging effect across many trees reduces their impact. For the training data parameter space on which our models were trained, refer to Table 1 and Table 2. For example, as shown by La Fazia et al., different power settings affect transmural lesion creation [26]. This is assumed not to be a concern with Random Forest as the effect of a different power setting is averaged in certain trees as long as the specific power setting is included in our training data parameter space. In addition, it has to be acknowledged that our models were trained on data exported from PVI procedures, while the data analysis included all atrial ablation procedures.

Thus, future studies using a similar machine learning approach must primarily address the below challenges. In particular, this means larger and more specific training datasets, for instance, only on PVI data, to enhance robustness and transferability. A refined model training process would also provide benefits, such as evaluating different ensemble learning techniques or neuronal networks.

In the second step of our approach, we analyzed the data from patients who underwent LI-guided ablation alongside our predicted AI and LSI metrics. While this is a retrospective study, data were provided anonymously during export. Thus, the potential for unmeasured confounders affecting the outcomes cannot be excluded. Our dataset represents a heterogeneous patient population, with a relatively small sample size of only 27 patients who underwent LI-guided ablation for different atrial arrhythmias. Likewise, data analysis was not conducted separately for different segments, such as anterior/superior or posterior/inferior. In addition, our dataset lacks periprocedural baseline characteristics, such as echocardiographic parameters, operator variability regarding experience and techniques used, and procedural characteristics such as procedural time. Also, our study did not examine the effect of different RF application durations on the mitigation of complications and long-term clinical outcomes. Thus, the potential for unmeasured confounders affecting the outcomes cannot be excluded, and the likelihood of statistical bias is also increased.

As already stated, the lower performance of our LSI prediction model leads to missing values for our LSI RF application duration. We, therefore, had to rely on MICE to deal with missing data. While we performed Little’s MCAR test and sensitivity analysis, we assumed that our data were not Missing Completely at Random (MCAR) and likely Missing at Random (MAR). This also comes with the assumption that our data were not Missing Not at Random (MNAR) as no other confirmation of MNAR, like comparing to different parameters like CF, was applicable. In addition to the already lower performance of our LSI model, this procedure may introduce bias. While the beforehand exclusion of lesions with RF application duration over 30 s and ILD over 6 mm admits established practices, similar aspects of bias are applicable.

As already acknowledged above, future studies would benefit from more specific and larger datasets that allow for subpopulation analysis, such as different types of arrhythmias or adjusting AI and LSI targets for anterior/superior and posterior/inferior segments.

## 5. Conclusions

This is the first retrospective cohort study with a comparative analysis comparing predicted AI and LSI metrics for LI-guided procedures. Our study reveals significant differences in median RF application durations depending on the lesion metric used. Moreover, the feasibility of using machine learning to predict lesion metrics was shown. Further studies are needed to assess different machine learning approaches and to evaluate the safety and efficacy of these results in a clinical setting.

## Figures and Tables

**Table 1:** The training parameters for the Ablation Index Model (the AI training dataset used).

Parameter	Median (IQR)
Contact Force, g·s	12.096(12.178)
Power, W	50.0 (9.8)
Ablation Index	362.259(196.058)

**Table 2:** The training parameters for the Lesion Size Index Model (the LSI training data set used).

Parameter	Median (IQR)
Contact Force, g·s	11.500(9.800)
Power, W	40.0(0.0)
RF current, mA	526.134(33.021)
Lesion Size Index	2.591(1.097)

**Table 3:** Performance metrics ($R^{2}$ and MAE) for the AI and LSI prediction models during cross-validation when applying a 30% split.

Model	R^2^	MAE
**AI prediction**		
Random Forest	0.972	6.617
Gradient Boosting	0.924	15.422
Ridge	0.807	41.044
Lasso	0.807	41.043
ElasticNet	0.807	41.044
**LSI prediction**		
Random Forest	0.940	0.132
Gradient Boosting	0.969	0.103
Ridge	0.925	0.163
Lasso	0.925	0.159
ElasticNet	0.925	0.161

**Table 4:** Patient demographics for the exported data used during data analysis, n=27 (LI-guided), are reported as count and percentage.

Patient Characteristics	
Age (years)	66±14.0 ^1^
Male, n (%)	12(43)
Ejection fraction ≤55%, n (%)	9(32)
Hypertension, n (%)	16(57)
Diabetes, n (%)	4(14)
Chronic kidney disease ^2^, n (%)	15(54)
Indication	
Atrial fibrillation (AF)	13 (48.2)
Atrial flutter (AFL)	12 (44.4)
Atrial tachycardia	2 (7.4)

^1^ M ± SD: Mean ± Standard Deviation. ^2^ KDIGO 2012 ≥ G2

**Table 5:** The main results with the lesion metrics (n=1090) for all lesions created. Continuous variables are reported as the median with inter-quartile range (IQR). The LI drop plateau was defined as the local minimum, and only the lesions that reached the operator’s desired LI-drop target were exported. AI and LSI targets were set to AI ≥400 and LSI ≥4.

Lesion Metric	Median (IQR)	*p*
RF application duration LI, s	11.92 (8.02–16.72) ^1^	<0.001 ^3^
RF application duration AI, s	7.32 (5.05–9.57) ^1^	<0.001 ^3^
RF application duration LSI, s	19.91 (17.59–22.95) ^1^	<0.001 ^3^
LI drop, Ω	20.36 (14.32–27.69)	-
LI start impedance, Ω	146.59 (135.96–158.96)	-
CF mean, g·s	18.18 (11.67–25.51)	-
Start CF, g·s	16.87 (10.26–24.23)	-
Ablation Index at LI plateau	476.46 (387.49–533.69) ^1^	-
Lesion Size Index at LI plateau	3.17 (2.47–3.55) ^1^	-
Interlesion distance, mm	3.66 (2.78–4.52) ^2^	-

^1^ Our machine learning models calculated the values. ^2^ Calculated using the k-nearest neighbor algorithm [21,22]. ^3^ p-values for Friedman test: $\chi^{2}\left(2\right)=1418.95$. Post-hoc Wilcoxon signed-rank test between different metrics, all $ps_{Bonferroni}<0.001$.

## Data Availability

The data presented in this study are available on request from the corresponding authors due to legal reasons.

## Abbreviations

The following abbreviations are used in this manuscript:

AF	Atrial fibrillation
AFL	Atrial flutter
AI	Ablation Index
CF	Contact force
GAMs	Generalized additive models
HPSD	high-power short-duration
ILD	Interlesion distance
LI	Local impedance
LSI	Lesion Size Index
MAE	Mean absolute error
Mean CV MSE	Mean squared error (MSE) from the coefficient of variation (CV)
MICE	Multiple imputation by chained equations
PV	Pulmonary vein
PVI	Pulmonary vein isolation
$R^{2}$	Coefficient of determination
RF	Radiofrequency
VIF	Variance inflation factors

## Appendix A

**Table A1:** Software library and versions used.

Python Library	Version
Numpy	1.26.4
Pandas	2.2.2
Scikit-learn	1.2.2
Scipy	1.11.4
Statsmodel	0.14.0
pyGAM	0.9.1
Miceforest	6.0.3
Matplotlib	3.7.1
Seaborn	0.12.2

**Table A2:** Hyperparameters and the value ranges used during the training process.

Model	Hyperparameters and Value Range
Random Forest	min_samples_split: 2,4,6
	min_samples_leaf: 1,2,3
Gradient Boosting	alpha: 0.001, 0.005, 0.01, 0.05, 0.1, 0.5, 0.99
Ridge	alpha: 0.001, 0.005, 0.01, 0.05, 0.1, 0.5, 0.99
Lasso	alpha: 0.001, 0.005, 0.01, 0.05, 0.1, 0.5, 0.99
ElasticNet	alpha: 0.001, 0.005, 0.01, 0.05, 0.1, 0.5, 0.99

## References

[1] Van Gelder I.C., Rienstra M., Bunting K.V., Casado-Arroyo R., Caso V., Crijns H.J.G.M., Potter T.J.R.D., Dwight J., Guasti L., Hanke T. (2024). 2024 ESC Guidelines for the Management of Atrial Fibrillation Developed in Collaboration with the European Association for Cardio-Thoracic Surgery (EACTS). Eur. Heart J..

[2] Calkins H., Hindricks G., Cappato R., Kim Y.-H., Saad E.B., Aguinaga L., Akar J.G., Badhwar V., Brugada J., Camm J. (2017). 2017 HRS/EHRA/ECAS/APHRS/SOLAECE Expert Consensus Statement on Catheter and Surgical Ablation of Atrial Fibrillation. Heart Rhythm..

[3] Chieng D., Segan L., Sugumar H., Al-Kaisey A., Hawson J., Moore B.M., Nam M.C.Y., Voskoboinik A., Prabhu S., Li L. (2023). Higher Power Short Duration vs. Lower Power Longer Duration Posterior Wall Ablation for Atrial Fibrillation and Oesophageal Injury Outcomes: A Prospective Multi-Centre Randomized Controlled Study (Hi-Lo HEAT Trial). EP Eur..

[4] Phlips T., Taghji P., El Haddad M., Wolf M., Knecht S., Vandekerckhove Y., Tavernier R., Duytschaever M. (2018). Improving Procedural and One-Year Outcome after Contact Force-Guided Pulmonary Vein Isolation: The Role of Interlesion Distance, Ablation Index, and Contact Force Variability in the ‘CLOSE’-Protocol. EP Eur..

[5] Venkatesh Prasad K., Bonso A., Woods C.E., Goya M., Matsuo S., Padanilam B.J., Kreis I., Yang F., Williams C.G., Tranter J.H. (2022). Lesion Index–Guided Workflow for the Treatment of Paroxysmal Atrial Fibrillation Is Safe and Effective—Final Results from the LSI Workflow Study. Heart Rhythm O2.

[6] Hussein A., Das M., Riva S., Morgan M., Ronayne C., Sahni A., Shaw M., Todd D., Hall M., Modi S. (2018). Use of Ablation Index-Guided Ablation Results in High Rates of Durable Pulmonary Vein Isolation and Freedom from Arrhythmia in Persistent Atrial Fibrillation Patients: The PRAISE Study Results. Circ. Arrhythm. Electrophysiol..

[7] Das M., Luik A., Shepherd E., Sulkin M., Laughner J., Oesterlein T., Duffy E., Meyer C., Jais P., Duchateau J. (2021). Local Catheter Impedance Drop During Pulmonary Vein Isolation Predicts Acute Conduction Block in Patients with Paroxysmal Atrial Fibrillation: Initial Results of the LOCALIZE Clinical Trial. EP Eur..

[8] Lian E., Pantlik R., Maslova V., Willert S., Moser F., Remppis A., Frank D., Demming T. (2024). Local Impedance Drop–Guided versus Lesion Size Index–Guided Pulmonary Vein Isolation. J. Interv. Card. Electrophysiol..

[9] Harris C.R., Millman K.J., van der Walt S.J., Gommers R., Virtanen P., Cournapeau D., Wieser E., Taylor J., Berg S., Smith N.J. (2020). Array Programming with NumPy. Nature.

[10] McKinney W. Data structures for statistical computing in python. Proceedings of the 9th Python in Science Conference.

[11] Pedregosa F., Varoquaux G., Gramfort A., Michel V., Thirion B., Grisel O., Blondel M., Prettenhofer P., Weiss R., Dubourg V. (2011). Scikit-Learn: Machine Learning in Python. J. Mach. Learn. Res..

[12] Virtanen P., Gommers R., Oliphant T.E., Haberland M., Reddy T., Cournapeau D., Burovski E., Peterson P., Weckesser W., Bright J. (2020). SciPy 1.0: Fundamental Algorithms for Scientific Computing in Python. Nat. Methods.

[13] Skipper S., Perktold J. statsmodels: Econometric and statistical modeling with python. Proceedings of the 9th Python in Science Conference.

[14] Servén D., Brummitt C., Abedi H. (2018). dswah/pyGAM.

[15] Hunter J.D. (2007). Matplotlib: A 2D Graphics Environment. Comput. Sci. Eng..

[16] Waskom M. (2021). seaborn: Statistical Data Visualization. J. Open Source Softw..

[17] Cartwright K.V. (2017). Simpson’s Rule Cumulative Integration with MS Excel and Irregularly-Spaced Data. J. Math. Sci. Math. Educ..

[18] Breiman L. (2001). Random Forests. Mach. Learn..

[19] Hastie T., Tibshirani R., Friedman J. (2009). The Elements of Statistical Learning.

[20] Bergstra J., Bengio Y. (2012). Random Search for Hyper-Parameter Optimization. J. Mach. Learn. Res..

[21] Fix E., Hodges J.L. (1989). Discriminatory Analysis—Nonparametric Discrimination: Consistency Properties. Int. Stat. Rev..

[22] Cover T.M., Hart P.E. (1967). Nearest Neighbor Pattern Classification. IEEE Trans. Inf. Theory.

[23] Kanamori N., Kato T., Sakagami S., Saeki T., Kato C., Kawai K., Chikata A., Takashima S., Murai H., Usu S. (2018). Optimal Lesion Size Index to Prevent Conduction Gap during Pulmonary Vein Isolation. J. Cardiovasc. Electrophysiol..

[24] Wita K., Dyrbuś M., Kowalski O., Wita M., Myrda K., Hoffmann A., Błachut A., Jadczyk T., Mizia-Stec K., Gołba K. (2024). The regional distribution of catheter ablations of atrial fibrillation and atrial flutter in Poland in 2023. Pol. Heart J..

[25] Winkle R.A. (2021). HPSD ablation for AF high-power short-duration RF ablation for atrial fibrillation: A review. J Cardiovasc Electrophysiol..

[26] la Fazia V.M., Pierucci N., Schiavone M., Compagnucci P., Mohanty S., Gianni C., della Rocca D.G., Horton R., Al-Ahmad A., di Biase L. (2024). Comparative effects of different power settings for achieving transmural isolation of the left atrial posterior wall with radiofrequency energy. Europace.

[27] Muffoletto M., Qureshi A., Zeidan A., Muizniece L., Fu X., Zhao J., Roy A., Bates P.A., Aslanidi O. (2021). Toward Patient-Specific Prediction of Ablation Strategies for Atrial Fibrillation Using Deep Learning. Front. Physiol..

[28] Ogbomo-Harmitt S., Muffoletto M., Zeidan A., Qureshi A., King A.P., Aslanidi O. (2023). Exploring interpretability in deep learning prediction of successful ablation therapy for atrial fibrillation. Front. Physiol..

[29] Razeghi O., Kapoor R., Alhusseini M.I., Fazal M., Tang S., Roney C.H., Rogers A.J., Lee A., Wang P.J., Clopton P. (2023). Atrial fibrillation ablation outcome prediction with a machine learning fusion framework incorporating cardiac computed tomography. J. Cardiovasc. Electrophysiol..

